# Development and validation of a scale to measure team communication behaviors

**DOI:** 10.3389/fpsyg.2022.961732

**Published:** 2022-12-08

**Authors:** Martina Hartner-Tiefenthaler, Ivana Loerinc, Sabina Hodzic, Bettina Kubicek

**Affiliations:** ^1^Institute of Management Science, Labor Science and Organization, Vienna University of Technology, Vienna, Austria; ^2^Faculty of Natural Sciences, Institute of Psychology, University of Graz, Graz, Austria

**Keywords:** remote working, flexible team, team communication, relational coordination, scale development, scale validation

## Abstract

**Introduction:**

With the COVID-19 pandemic, remote work was increased all over the globe. As a consequence, workers had to adapt their communication behaviors to smoothly coordinate work in their flexible teams (i.e., when team members divide work between the office and their homes). Drawing on relational coordination theory, we constructed and validated a scale to capture the most relevant team communication behaviors.

**Methods:**

We employed interviews and focus groups to construct the scale, refined the scale based on three samples with employees working flexibly and finally validated the scale with 130 teams from diverse organizations.

**Results:**

Our scale comprises three dimensions: focused communication, knowledge sharing and spontaneous communication. All three dimensions showed convergent validity with team planning and discriminant validity with time-spatial flexibility. Also, predictive validity with collective efficacy and team viability was achieved for focused communication and knowledge sharing. Spontaneous communication only predicted collective efficacy, but not team viability.

**Discussion:**

We conclude that the TCS is a reliable and valid measure for assessing team communication and contribute by focusing on behaviors.

## Introduction

The current COVID-19 pandemic has caused a boost of remote work all over the globe. Previously, remote work was the exception from the norm and needed to be particularly negotiated with the supervisor (Gajendran et al., 2015). With the pandemic and the accompanying change of working methods, remote work has become the “new normal” and office workers moved their work mainly or even entirely to their homes (International Labour Organization, 2020). This sudden switch to remote work resulted in positive as well as negative consequences for individual workers and teams. Although workers have appreciated the gained autonomy and flexibility (Gajendran and Harrison, 2007; Allen et al., 2015), the challenge for team communication is evident: due to the lack of face-to-face contact, spontaneous communication in the office, at the coffee corner or during lunch hours is lacking, which potentially impairs information and knowledge sharing among team members (Hinds and Mortensen, 2005). Therefore, it is important to shed further light on team communication behaviors and assess how it can be evaluated and managed best for effective work, especially in the context of work flexibility where individual team members independently choose when and where to work (Putnam et al., 2014). As we are interested on the team level, we define this work context as flexible teams.

There is growing evidence that flexible teams and the accompanying lack of co-presence in the office might impair interpersonal processes and knowledge sharing among team members (Allen et al., 2015; van der Meulen et al., 2019) and that spontaneous communication might buffer the negative effects (Hinds and Mortensen, 2005). Only recently, the role of unplanned communication in the office has been emphasized (e.g., Methot et al., 2021; Puranik et al., 2021). However, no existing measure captures knowledge sharing behaviors in teams or assesses spontaneous communication between team members. Most of the measures of team communication so far focus either on assessing the frequency (and/or quantity) of communication and knowledge sharing (e.g., Bunderson and Sutcliffe, 2003; Fonner and Roloff, 2012; Kessel et al., 2012) or the quality of communication (e.g., Hoegl and Gemuenden, 2001; González-Romá and Hernández, 2014). Although, quality of communication is more strongly related to team performance than frequency of communication (Marlow et al., 2018), existing communication quality measures either assess attitudes or satisfaction with the communication between team members and not the actual quality of communication behaviors. Moreover, only one measure so far assesses team behaviors (Fisher, 2014). However, the focus of this measure is on team coordination, rather than on team communication. Focusing on team communication behaviors would allow teams to optimize their communication strategies. Another important aspect of previous measures is that the reference point is usually on the individual level (items are individually formulated) and not on the team level, which decreases the ratings’ representativeness for the entire team (Klostermann et al., 2021).

The lack of team communication measures is in contrast to the importance of communication in interdependent teams as highlighted by relational coordination theory (Gittell, 2000,a,b, 2016; Gittell et al., 2008, 2010; Gittell and Ali, 2021). Relational coordination theory is a multi-level theory that describes coordination in interdependent teams. We draw on this theory as it is particularly suitable for an uncertain context such as teams in which employees are able to choose flexibly when and where to work (Wessels et al., 2019). This flexibility, though positive at the individual level, makes it uncertain and unpredictable for team members whether they will meet their co-workers at the office. In contrast to fully co-located or fully virtual teams, face time among team members must be purposefully organized and cannot be taken for granted in flexible teams, amplifying the challenges of communication in the teams (Waerzner et al., 2017). Even the team members who might choose to work in the office themselves are affected when their co-workers opt to work remotely and they have to adapt their communication behaviors nonetheless. Furthermore, when a considerable number of co-workers works remotely, more and more team members might then decide to also work from home as the incentive to work onsite declines when the office is half-empty (i.e., contagion effect, Rockmann and Pratt, 2015). Therefore, we consider it important to draw attention towards team communication behaviors as the lack of direct communications in the office leads to social and professional isolation, obstructing knowledge sharing (Cleveland and Ellis, 2015) and hampering social relationships and bonds at work (Gajendran and Harrison, 2007). Since the prevalence of time-spatial flexibility for workers has increased recently and as such flexible teams have become common across the globe, it is highly relevant to particularly focus on this uncertain team context and consider the so far neglected aspects of team communication behaviors.

Drawing on relational coordination theory literature (Gittell et al., 2010; Gittell, 2011b, 2016) as well as literature from the remote work context (Hinds and Mortensen, 2005; Methot et al., 2021; Puranik et al., 2021), we developed and validated the team communication scale (TCS). Based on qualitative and quantitative studies, we propose a three-dimensional structure of the TCS, with focused communication, knowledge sharing, and spontaneous communication as dimensions. Subsequently, we validate the TCS using a sample of 130 flexible teams from diverse organizations.

The TCS contributes to existing literature in at least three ways. First, in contrast to previous measures, the TCS captures team communication behaviors and does not focus on satisfaction with communication, which rather measures attitudinal aspects. Also, it focuses on the quality rather than the less relevant aspect of frequency of communication (Shockley et al., 2021). Second, the TCS is suitable for all types of teams, such as remote teams, co-located teams, virtual teams or hybrid teams, but especially for flexible teams. The items were developed through interviews with employees who work flexibly in order to address the changes of work brought about by increased digitalization and flexibilization. However, the items are formulated in a general way, which makes the scale suitable for any type of team. Finally, the TCS encompasses spontaneous communication, which is an aspect of team communication that has been neglected in previous instruments so far. Spontaneous communication is of great relevance especially for flexible teams, as it fosters information exchange between team members and builds positive social emotions (Hinds and Mortensen, 2005; Methot et al., 2021).

## Team communication and workplace flexibility

Traditionally, literature on workplace flexibility that encompasses flexibility about when and where work is conducted (Putnam et al., 2014), has mainly focused on the individual or the organizational level and neglected the team level (Raghuram et al., 2019). There is unequivocal evidence that remote working positively relates to job satisfaction due to the underlying autonomy provided to employees (Gajendran and Harrison, 2007; van der Lippe and Lippényi, 2020) and also improves the reconcilability between work and private life (Allen et al., 2013). However, when it comes to team outcomes, relations are more ambiguous and point toward challenges with regard to knowledge sharing (Golden and Raghuram, 2010) and spontaneous communication (Waerzner et al., 2017). Face time is reduced in flexible teams and dependence on digital communication for coordination (e.g., e-mails, collaborative software, etc.) is more prevalent. While standard operating procedures are hardly affected, the change in communication of flexible teams might lead to impaired mutual adjustments between co-workers (Waerzner et al., 2017). Thus, teams must adapt their communication routines (Waerzner et al., 2016) and use communication media that are appropriate for their tasks (Dennis et al., 2008) to ensure performance.

To conceptualize communication in flexible teams, we refer to relational coordination theory (Gittell et al., 2008, 2010; Havens et al., 2010). Relational coordination theory (Gittell, 2011b, 2016) is a multi-level theory and describes coordination in interdependent work groups in uncertain contexts. It acknowledges the importance of direct exchanges between team members in addition to the formal organizational structure (Gittell and Douglass, 2012). Relational coordination is defined as “a mutually reinforcing process of interaction between communication and relationships carried out for the purpose of task integration” (Gittell, 2002, p. 301). To effectively coordinate work and function as a team, team members build reciprocal relationships that are informal and not deliberately created or prescribed. These relationships emerge through informal communication and the shared experiences of team members (Gittell and Douglass, 2012). In line with relational coordination theory, effective team communication was found to be a key factor for success in flexible teams (Shockley et al., 2021). In the following, we therefore discuss which aspects of communication are particularly relevant for the flexible team context.

## Dimensions of team communication

Relational coordination theory (Gittell, 2000, 2016; Gittell et al., 2010) considers timely, accurate, and solution-oriented communication as particularly relevant for team functioning. In teams where face time is rare, timely and accurate communication becomes even more relevant, since nonverbal communication cues that would normally provide contextual information during face-to-face discussions are missing. Also, when working remotely, it might be difficult to ascertain when to contact someone (in order not to disturb him or her) as well as whether information has been understood accurately because social cues or direct feedback are missing. In addition to timely and accurate communication, relational coordination theory suggests taking the frequency of communication into account. However, existing research shows that the frequency of communication is less important (Shockley et al., 2021) for team performance than the quality of communication (Marlow et al., 2018), which also plays an important role in reducing stress. Second, frequent communication might be a double-edged sword in today’s work context, as a high number of messages can lead to information overload (i.e., e-mail spamming; Kalman and Ravid, 2015; Stich et al., 2018) or encourage the extension of working hours (e.g., autonomy paradox, Mazmanian et al., 2013), which is likely to have detrimental effects for workers’ well-being (Schlachter et al., 2017) and might also hamper performance in the long run due to a lack of recovery (Sonnentag, 2003). Therefore, we argue that workers in today’s work teams are challenged to filter irrelevant information (Fay, 2011). Due to this reason, communication needs to be focused, timely and accurate and therefore we denominate our first dimension as focused communication. Focused communication is defined as the behavioral act of exchanging accurate information with regard to tasks, time or responsibilities among team members. When focused communication cannot be taken for granted, it creates a major challenge for team communication (Waerzner et al., 2016).

In addition to how communication is carried out between team members, relational coordination theory (Gittell, 2011b) also addresses the aspect of what kind of communication is exchanged such as sharing knowledge among team members. Knowledge sharing is seen as an important indicator for building high quality relationships in teams and refers to informal and free-flowing cooperative exchanges among team members (Golden and Raghuram, 2010). It requires not only the interaction with co-workers, but also the willingness for exchanging knowledge (Cross et al., 2001; Golden and Raghuram, 2010), which is strongly influenced by the relational qualities of remote workers (Golden and Raghuram, 2010). Overall, knowledge sharing constitutes a crucial element for organizational success in today’s business environment (Cross et al., 2001; Hansen et al., 2005; Ben-Menahem et al., 2016) and is considered to be relevant for innovation in flexible teams (Gajendran and Joshi, 2012), but also facilitates learning and standard work processes (Brown and Duguid, 1991; Chiaburu and Harrison, 2008). Facilitating knowledge and information exchange was an important buffer of stress, especially stress imposed by technology usage for remote working during the COVID-19 pandemic (Zito et al., 2021). Sharing knowledge also relates to active problem-solving (Gittell, 2011b). However, in flexible teams, it might be less clear whom to contact when a problem needs to be solved immediately, as a quick question in the shared office space is not possible anymore (Waerzner et al., 2017).

To serve the particular context of flexible teams, we extend the aspects described by relational coordination and draw attention towards spontaneous communication, as the third dimension of the TCS. Spontaneous communication refers to unplanned, informal interactions that spontaneously take place when meeting colleagues unexpectedly, for example at the coffee corner or in the hallway. This type of communication is particularly challenging in flexible teams (Waerzner et al., 2016) as casual encounters between team members are less likely (Raghuram, 1996; Golden and Raghuram, 2010). When team members work at different times and locations, communication becomes more complex (Te’eni, 2001) and takes additional effort such as planning (Prem et al., 2021). In flexible teams, talking to a colleague about a specific topic or just asking a short question relies on digital media, which might only be used for factual communication and rarely for casual exchanges. Hence, the more team members work from home, the less likely informal interactions are (van der Lippe and Lippényi, 2020). However, a certain level of informal interactions is important for team functioning when working from home (Windeler et al., 2017). When co-workers spontaneously exchange information about current events, they may also share information considered less relevant (that they would not share at all *via* digital media) although it may prove relevant at a later point in time. Moreover, spontaneous communication facilitates the creation of shared context (Hinds and Mortensen, 2005) and decreases the probability of misunderstandings and conflicts in the team (Cramton, 2001). Based on these arguments, we expand the relational coordination framework and consider *spontaneous communication* as a necessary dimension for our TCS in addition to *focused communication* and *knowledge sharing*.

## Scale development

**Figure 1:**
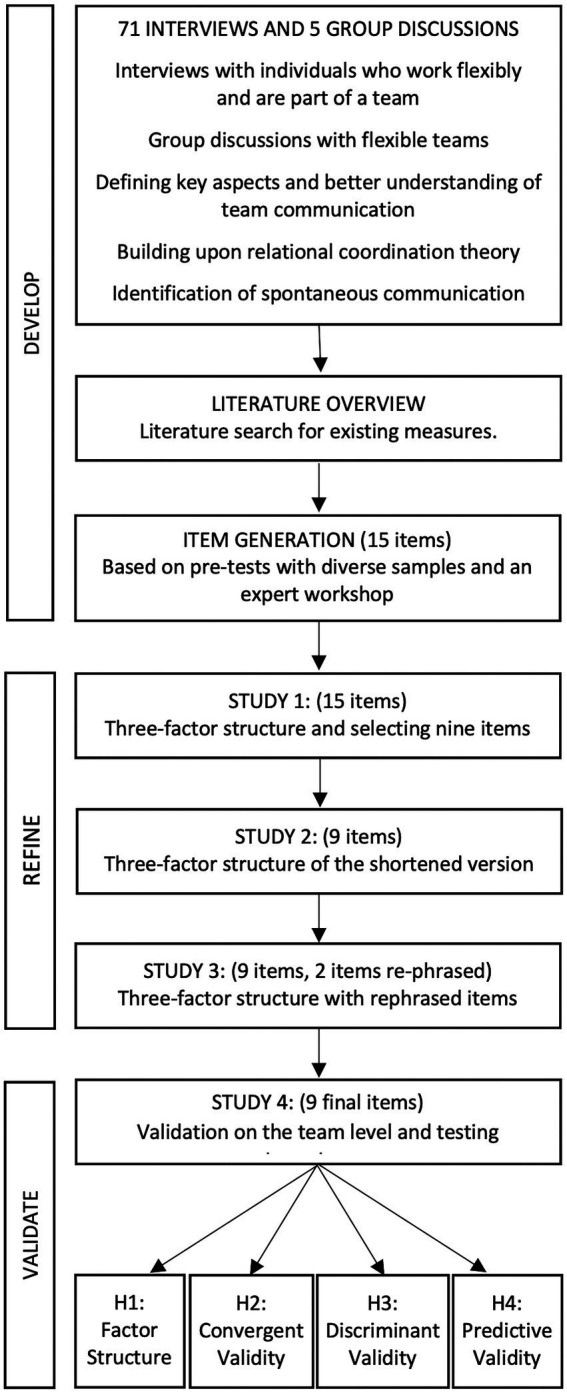
Development and validation of TCS.

The goal of this paper is to develop, refine and validate a scale that captures the behavioral aspects of team communication. The development and validation process was iterative and comprised several steps, including qualitative and quantitative data collection from individuals and teams experiencing workplace flexibility (see Figure 1).

To gain a deeper understanding of the specific context of flexible teams and their communication processes, we conducted 71 interviews with individuals from different organizational contexts (IT, public administration, consulting) having time-spatial flexibility and being members of a team. Using the critical incidence technique (Flanagan, 1954; Butterfield et al., 2005), we asked interviewees to describe their work context and narrate situations where they faced challenges in team communication. Additionally, five group discussions with existing flexible teams from the IT context were conducted in the frame of a master thesis supervised by the first author (Vecerka, 2019). This allowed us to examine communication processes among flexible teams whose members use workplace flexibility. The interviews and the group discussions were transcribed and then analyzed to identify relevant dimensions of team communication and supported the dimensions derived from relational coordination theory (Gittell, 2011b), such as focused communication and knowledge sharing and suggested adding the dimension of spontaneous communication.

In formulating the items, we drew on existing knowledge and searched for available scales capturing focused communication, knowledge sharing or spontaneous communication. With regard to focused communication and knowledge sharing we mostly relied on items used in relational coordination theory (Gittell, 2011a; Gittell and Ali, 2021). For spontaneous communication, we primarily found qualitative studies further supporting the relevance of this dimension for the TCS (Holmes and Marra, 2004; Oertig and Buergi, 2006; Elsbach et al., 2010; Fay, 2011; Fay and Kline, 2011). Inspired by Hinds and Mortensen (2005), we did not ask about the content of spontaneous communications, but rather were interested in whether team members had unplanned, spontaneous interactions within the team.

In the first stage of the scale development, we conducted an expert workshop to formulate items that were tested in pre-tests in four organizations from different industries (architecture *n* = 270; labor union, *n* = 243, information technology, *n* = 44, and telecommunication, *n* = 162) resulting in data from 719 employees. The aim of the pre-test was to assess the applicability of the generated items across various sectors. We compared the item factor loadings from an exploratory factor analysis conducted with datasets from each organization and selected a set of 15 items for further validation (see Table 1) representing the three dimensions - *focused communication*, *spontaneous communication*, and *knowledge sharing*. All items referred to the team level (e.g., *“In our team, we communicate in a timely manner”*) and asked for agreement from 1 (*strongly disagree*) to 5 (*strongly agree*). Furthermore, all items were worded positively, to ease the understanding and avoid the creation of an artificial factor (Dalal and Carter, 2014).

**Table 1:** Study1: Item wording, descriptive statistics, Cronbach’s alpha, factor loadings from exploratory factor analysis.

Code	Item	Study 1					
		*α*	*M*	*SD*	Latent factor			1	2	3
*Focused communication*		0.85	3.76	0.73			
FC1	… we communicate in a timely manner.		3.54	0.98	0.45	0.07	0.27
FC2	… we communicate exactly and precisely.		3.50	0.96	0.98	0.00	−0.17
FC3	… we communicate result-oriented.		3.87	0.93	0.77	−0.04	0.02
FC4*	… we communicate frequently.		3.94	0.91	0.44	0.25	0.14
FC5*	… we discuss problem solutions.		4.08	0.82	0.61	0.13	0.01
*Knowledge sharing*		0.83	3.92	0.66			
KS1	… we keep each other posted.		3.88	0.88	−0.04	0.00	0.90
KS2	… we know about the work of other team members.		3.82	0.83	0.03	0.07	0.63
KS3	… we know the expertise of other team members.		3.92	0.86	0.15	0.08	0.43
KS4*	… we consult each other regularly.		3.92	0.94	0.36	−0.08	0.53
KS5*	… it is clear who can help you with which problem.		4.09	0.81	0.11	0.11	0.46
*Spontaneous communication*		0.70	3.84	0.61			
SC1	… we come up with solutions through spontaneous conversations.		3.48	0.94	0.15	0.48	−0.17
SC2	… we also discuss things spontaneously.		4.10	0.79	−0.24	0.88	0.01
SC3	… we communicate on a short notice.		3.99	0.85	0.10	0.53	−0.02
SC4*	… we solve problems in passing.		3.49	1.07	−0.00	0.69	−0.22
SC5*	… we have opportunities for quick exchanges.		4.11	0.85	0.22	0.35	0.10

*Items deleted after Study 1.

## Scale validation

### Factor structure

Although the suggested dimensions are likely to be interrelated with each other, we assume that focused communication, knowledge sharing and spontaneous communication are separated from each other and follow a three-factor structure (Hypothesis 1).

### Convergent validity

We expect that team communication positively correlates with other constructs important for team effectiveness, such as *team planning* (DeChurch and Haas, 2008; Fisher, 2014). Team planning, a necessary team process for successful team functioning (Fisher, 2014), is defined as the “degree to which the team arrives at an effective initial plan of behavioral action” (Ilgen et al., 2005, p. 523). For successful team planning it is necessary to first gather information and then use this information to develop a plan for attaining goals (Ilgen et al., 2005). Thus, we argue that focused communication and knowledge sharing helps to build up the knowledge necessary to make effective plans. Since spontaneous communication among team members was found to support the creation of a shared context (Hinds and Mortensen, 2005), we assume that this also makes planning on the team level a more fluid process. We therefore hypothesize that all three TCS dimensions *focused communication* (Hypothesis 2a), *knowledge sharing* (Hypothesis 2b) and *spontaneous communication* (Hypothesis 2c) will be positively related to team planning.

### Discriminant validity

Although our TCS is particularly relevant for flexible teams, where each member can choose (at least to some extent) where and when to work, we consider all three dimensions as distinct constructs from workplace flexibility (Putnam et al., 2014). Making use of *time-spatial flexibility* (Shockley and Allen, 2007; Wessels et al., 2019) represents the context in which a specific behavior (i.e., team communication) is executed. We argue that this contextual circumstance amplifies the importance of the three dimensions of the TCS, but does not increase their likelihood. To test for discriminant validity, we propose that all three dimensions of the TCS, *focused communication* (Hypothesis 3a), *knowledge sharing* (Hypothesis 3b) and *spontaneous communication* (Hypothesis 3c) are only weakly related to time-spatial flexibility.

### Predictive validity

In order to test the predictive validity of our scale, we investigate the relationship between the three dimensions of the TCS and two important team outcomes, namely collective efficacy and team viability. Collective team efficacy is defined as teams’ beliefs about their capability to perform tasks successfully (Bandura, 1997; Salanova et al., 2003). It has been shown to influence the levels of collective well-being and performance and acts as an important buffer against job demands and stress (Salanova et al., 2003). Even though antecedents of collective efficacy have been investigated less frequently than its clear link with team performance, it is assumed that it evolves through team observable behaviors, such as team interactions and information exchange (Tasa et al., 2007). This is in line with Marks et al. (2001) who argued that collective efficacy, as an emergent state, is influenced by interdependent team processes, among which communication plays a key role. We therefore hypothesize that all three TCS dimensions *focused communication* (Hypothesis 4a), *knowledge sharing* (Hypothesis 4b) and *spontaneous communication* (Hypothesis 4c) will be positively related to *collective efficacy*.

*Team viability* refers to the willingness of team members to continue working together (Sundstrom et al., 1990). It is seen as an important outcome of team processes in virtual teams (Marlow et al., 2017), as well as a good and relevant indicator of future team effectiveness when teams undergo changes (Bell and Marentette, 2011). In line with this, Ortega et al. (2010) showed that team learning behavior, which includes aspects of team communication (such as question asking, seeking feedback, reflecting on results, and discussing errors or unexpected situations), significantly predicted team viability in virtual teams. Additionally, Foo et al. (2006) found that open communication, which encompasses knowledge sharing, is positively related to team viability. Spontaneous communication has been shown to be especially important in distributed teams because it attenuates potential conflicts in distributed teams (Liu et al., 2020) by allowing team members to share information and build a common shared context and understanding (Hinds and Mortensen, 2005). We assume that teams with fewer conflicts and more shared context are more likely to want to continue working in their team. We therefore hypothesize that all three TCS dimensions *focused communication* (Hypothesis 5a), *knowledge sharing* (Hypothesis 5b) and *spontaneous communication* (Hypothesis 5c) will be positively related to *team viability*.

## Materials and methods

### Study 1: Three-factor structure and reducing scale to nine items

As survey length commonly poses a challenge in organizational research, we decided to use Study 1 not only to evaluate the proposed three-factor-structure of the TCS, but also to shorten the scale to three items per dimension.

#### Sample

In 2018, we collected data from workers in an architecture organization, who wanted to evaluate their team communication. All 416 employees were invited to participate and *n*_1_ = 323 completed the questionnaire, resulting in a 78% response rate. Overall, 33 percent of the participants were female, their mean age was 37.6 years (*SD* = 11.67) and in average, they had worked in their organization for 7.1 years (*SD* = 8.01). Of the participants, 68.1% held an academic degree.

#### Measures

Team communication behavior with its three dimensions, focused communication, knowledge sharing, and spontaneous communication, was measured with the 15 items developed based on the qualitative interviews, group discussions and available published scales. Participants were asked to indicate their agreement to statements about team communication behaviors (“In our team…”) on a 5-point Likert scale from 1 (strongly disagree) to 5 (strongly agree).

#### Results

We conducted an exploratory factor analysis in Mplus Version 8 (Muthén and Muthén, 2017). Bartlett’s test of sphericity was significant (*χ*^2^ (105) = 2031.830, *p* < 0.001) and the Kaiser-Meyer-Olkin (KMO) measure of sampling adequacy indicated strong relationship among individual variables (KMO = 0.907), showing that the collected data were suitable for factor analysis (Hair et al., 1995). As we expected the three factors to be correlated, we chose an oblique factor rotation – Geomin (Yates, 1987). The change of chi-square values between models with one (*χ*^2^_90_ = 440.70), two (*χ*^2^_76_ = 247.07), and three factors (*χ*^2^_63_ = 134.54) was significant at *p* < 0.01, supporting our proposed three-factor-structure. The Cronbach’s alpha of the dimensions is presented in Table 1. Subsequently, we aimed for three items per scale and selected items per dimension based on their factor loadings, cross-loadings and phrasing (see Table 1). For the scale *focused communication*, we selected three items that showed the most suitable fit. One item was not selected as a closer analysis revealed that the wording was more task than communication oriented. For the scale *knowledge sharing* three items were selected based on the highest factor loadings and lack of pronounced cross-loadings on the remaining factors. In addition to factor loadings, we decided to drop one item of the scale *spontaneous communication* despite suitable factor loadings due to its German colloquial phrasing. We considered it as a possible disadvantage for translation and regional differences. Based on Study 1, nine items were selected for further validation (see Table 1).

### Study 2: Three-factor structure of the shortened version

In the next step of our analysis, we used data collected from individual workers to support the factor structure and selection of the final nine items. In cooperation with the Chamber of Labor in Lower Austria (an organization which represents all employed workers in that region), between 2018 and 2019, we invited a random sample of approximately 10,000 workers to participate in a paper-pencil survey. Our goal was to attract a more diverse sample of participants since in most studies higher educated people are over-represented. However, we are aware that self-selection bias is still probable (Søgaard et al., 2004).

#### Sample

In total, 838 workers completed the survey, but only *n*_2_ = 792 indicated to be working in a team and were, thus, used in the subsequent analysis. About half of the participants (55.0%) were female, their mean age was 42.7 years (*SD* = 10.97) and on average they had worked in their organization for 12.2 years (*SD* = 10.30). The education level of participants was balanced, as only 25.6% held an academic degree and 33.6% completed high school.

#### Measures

In Study 2, team communication behavior with its three dimensions (focused communication, knowledge sharing, and spontaneous communication) was measured with the reduced scale of nine items. Participants were asked to indicate to which extent the statements apply to their team on a 5-point Likert scale, ranging from 1 (strongly disagree) to 5 (strongly agree).

#### Results

To assess the model fit, we conducted a confirmatory factor analysis (CFA) in Mplus (Muthén and Muthén, 2017) using a maximum likelihood estimator with Satorra–Bentler scaled chi-square statistic (MLR in Mplus; Satorra and Bentler, 1994) which is robust against non-normally distributed data. Both Bartlett’s test of sphericity (*χ*^2^ (36) = 3721.510, *p* < 0.001) and the KMO measure of sampling adequacy (KMO = 0.879), supported the application of CFA on the collected data (Hair et al., 1995). Even though the TCS items are measured by a 5-point Likert scale, and are thus ordinal in nature, we used a maximum likelihood estimator, as ordinal data with five and more categories can be treated as continuous for the purposes of CFA analysis (Rhemtulla et al., 2012). Both incremental (comparative fit index − CFI, Tucker-Lewis index − TLI) and absolute (root mean square error of approximation − RMSEA and standardized root mean residual − SRMR) fit indices were calculated. The three-factor model yielded an acceptable fit with the data, *χ*^2^_24_ = 124.44, CFI = 0.96 and TLI = 0.95, RMSEA = 0.07 and SRMR = 0.04 (Bentler, 1992; Hu and Bentler, 1999; Vandenberg and Lance, 2000). The internal consistency of all three subscales was also within acceptable limits with Cronbach’s alphas ranging between 0.76 and 0.86 (see Table 2, Nunnally and Bernstein, 1994).

**Table 2:** Study 2 and 3: Item wording, descriptive statistics, Cronbach’s alphas and standardized factor loadings from confirmatory factor analysis (individual level).

Code	Item	Study 2						Study 3					
		*α*	*M*	*SD*	Latent factor			*α*	*M*	*SD*	Latent factor			1	2	3	1	2	3
*Focused communication*		0.86	3.64	0.92				0.86	3.79	0.89			
FC1	… we communicate in a timely manner.		3.71	1.02	0.82				3.79	1.01	0.79		
FC2	… we communicate exactly and precisely.		3.48	1.05	0.86				3.72	1.04	0.86		
FC3	… we communicate result-oriented.		3.75	1.06	0.79				3.90	0.98	0.83		
*Knowledge sharing*		0.76	3.94	0.83				0.85	4.01	0.84			
KS1	… we keep each other posted.		3.99	0.97		0.79			4.05	0.98		0.82	
KS2*	… we know about the work of other team members.		3.86	1.03		0.67			---	---		---	
KS3*	… we know the expertise of other team members.		3.98	1.04		0.67			---	---		---	
KS2**	… we proactively inform the others about relevant news.								4.04	0.93		0.79	
KS3**	… we exchange our knowledge and experiences.								3.95	0.99		0.81	
*Spontaneous communication*		0.85	3.84	0.89				0.85	3.96	0.86			
SC1	… we come up with solutions through spontaneous conversations.		3.47	1.10			0.72		3.78	1.05			0.83
SC2	… we also discuss things spontaneously.		3.98	1.03			0.89		4.02	0.99			0.84
SC3	… we communicate on a short notice.		4.11	0.91			0.85		4.14	0.89			0.77

*Items rephrased after Study 2. **Items used in Study 3 and Study 4.

### Study 3: Three-factor structure with rephrased items

Based on an expert workshop, we concluded that two items from the scale knowledge sharing (KS2 and KS3) were not sufficiently behavior-oriented, but were rather passively describing an emergent state. Therefore, a decision was made to rephrase the two items (see Table 2). To test the suitability of the rephrasing, we conducted another survey in cooperation with the Chamber of Labor in Lower Austria in 2020. Sampling was based on the same principle as in Study 2, using a random sample of employees in Lower Austria: approximately 10,000 workers were invited to participate in the study. To economize on entering paper-pencil surveys, we sent out postcards with the link to the online survey. Additionally, we used several mailing lists to increase the number of participants and further spread the survey among workers.

#### Sample

Of the 601 workers, who completed the survey, *n*_3_ = 515 were included in our analysis, as they answered all nine items of the TCS and also indicated to be working in a team. Similar to Study 2, about half of the participants (54.8%) were female. Their mean age was 39.6 years (*SD* = 10.49) and on average, they had worked 8.8 years (*SD* = 8.65) in their current organization. Of the participants, 39.0% held an academic degree and 31.4% completed high school.

#### Measures

We measured team communication using the three dimensions of the TCS and rephrasing two items of knowledge sharing in order to capture behaviors (see Table 2). As previously, participants were asked to indicate to which extent the statements apply to their team on a 5-point Likert scale, ranging from 1 (strongly disagree) to 5 (strongly agree).

#### Results

Analogous to Study 2, we conducted a confirmatory factor analysis (CFA) in Mplus 8 (Muthén and Muthén, 2017). Bartlett’s test of sphericity (*χ*^2^ (36) = 2884.608, *p* < 0.001) and the KMO measure of sampling adequacy (KMO = 0.926) showed that the collected data was suitable for CFA (Hair et al., 1995). Data showed a good fit for the three-factor model with *χ*^2^_24_ = 56.80, CFI = 0.98, TLI = 0.97, RMSEA = 0.05 and SRMR = 0.02 (Bentler, 1992; Hu and Bentler, 1999; Vandenberg and Lance, 2000). We compared the fit of the three-factor model with a one-factor model and three two-factor models to assess the best fitting model (Byrne, 2012). In each step, the change in *χ*^2^ was significant, thus implying that additional factors improved the model fit (see Table 3). An acceptable model fit was already achieved by the two-factor model where the dimensions focused communication and knowledge sharing were combined into one factor (*χ*^2^_26_ = 77.97, *χ*^2^/*df* = 3.00, CFI = 0.97 and TLI = 0.96, RMSEA = 0.06, SRMR = 0.03). However, after the introduction of the third factor into the model, a significant change in *χ*^2^ (∆ *χ*^2^_2_ = 21.77) was observed, thus supporting our hypothesized three-factor structure of the TCS. The three-factor model fit the data well with *χ*^2^_24_ = 54.13, *χ*^2^/df = 2.26, CFI = 0.99 and TLI = 0.98, RMSEA = 0.05 and SRMR = 0.02. Cronbach’s alphas of focused communication (*α* = 0.86) and spontaneous communication (*α* = 0.85) remained acceptable (Nunnally and Bernstein, 1994) and for knowledge sharing it increased by 0.09 to *α* = 0.85, compared to Study 2, supporting the better fit of the rephrased items.

**Table 3:** Study 3: Confirmatory factor analysis on the individual level.

*Model*	*χ^2^*	*df*	*χ^2^/df*	∆ *χ^2^*	∆ *df*	*CFI*	*TLI*	*RMSEA*	*SRMR*
One-factor model	237.46*	27	8.79	1648.99*	9	0.89	0.86	0.12	0.06
Two-factor model									
1^st^ factor: focused & spontaneous communication 2^nd^ factor: knowledge sharing	231.65*	26	8.91	4.72*	1	0.89	0.85	0.12	0.06
1^st^ factor: knowledge sharing and spontaneous communication 2^nd^ factor: focused communication	193.27*	26	7.43	29.56*	1	0.91	0.88	0.11	0.05
1^st^ factor: focused communication and knowledge sharing 2^nd^ factor: spontaneous communication	77.97*	26	3.00	99.47*	1	0.97	0.96	0.06	0.03
Three-factor model	54.18*	24	2.26	21.77*	2	0.98	0.98	0.05	0.02

*p < 0.001. The Chi-square change was calculated by a scaling correction because the chi-square values for MLR cannot be compared directly (Muthén and Muthén, 2017). All two-factor models were compared to the one-factor model. The three-factor model was compared to the best fitting two-factor model (i.e., 1^st^ factor: spontaneous communication; 2^nd^ factor: focused communication & knowledge sharing).

### Study 4: Validation on the team level and testing hypotheses

#### Sample

Finally, in Study 4, data was collected between 2019 and 2022 from teams that worked flexibly to some extent using purposive sampling. The participants were able to choose between the German and English version of the questionnaire. We contacted team leaders *via* two basic routes: (1) we published press releases to attract team leaders from various organizations to participate in the study with their team members and (2) we continuously asked our personal contacts or encouraged students to help us with data collection in the frame of their master theses or for course credits (5%). To incentivize participation for team leaders, each team received feedback indicating the aggregated team results including benchmarks comparing them to other teams. For the validation of the TCS, we included only teams where at least three team members completed the survey. The final dataset included 677 individuals belonging to 130 flexible teams, ranging in size between 3 and 22 team members (*M* = 5.21 team members, *SD* = 2.34). The mean age of the participants was 36.7 years (*SD* = 15.53). With regard to gender, 44.5% were female, 52.0% male and 3.5% of the participants did not indicate their gender. Furthermore, 65.1% held an academic degree.

#### Measures

See Table 4 for descriptive statistics, Cronbach’s alphas, and correlations of all measures and Table 5 for the final list of TCS items.

**Table 4:** Study 4: Descriptive statistics, Cronbach’s alphas, correlations, ICCs and AVE.

*Scale*	*α*	*M*	*SD*	*ICC1*	*ICC2*	*AVE*	$\sqrt{AVE}$	1	2	3	4	5	6
1. Focused communication	0.91	3.98	0.46	0.18	0.54	0.69	0.83						
2. Knowledge sharing	0.90	4.24	0.42	0.17	0.53	0.64	0.80	0.75*					
3. Spontaneous communication	0.87	4.18	0.42	0.17	0.51	0.67	0.82	0.58*	0.66*				
4. Team planning	0.83	3.70	0.40	0.16	0.50	0.41	0.64	0.62*	0.56*	0.43*			
5. Time-spatial flexibility	0.85	3.33	0.67	0.28	0.67	0.52	0.72	0.08	0.05	−0.03	0.12		
6. Collective efficacy	0.94	4.57	0.32	0.21	0.58	0.68	0.83	0.65*	0.67*	0.61*	0.57*	0.06	
7. Team viability	---	4.54	0.43	0.20	0.56	---	---	0.66*	0.69*	0.54*	0.61*	0.01	0.66*

*Significant at *p* < 0.001.

**Table 5:** Study 4: Confirmatory factor analysis on the team level.

*Model*	*χ^2^*	*df*	*χ^2^/df*	∆ *χ^2^*	*∆ df*	*CFI*	*TLI*	*RMSEA*	*SRMR*
One-factor model	511.77*	27	18.95		9	0.81	0.75	0.16	0.09
Two-factor model									
1^st^ factor: focused & spontaneous communication 2^nd^ factor: knowledge sharing	436.47*	26	16.79	45.65*	1	0.84	0.78	0.15	0.08
1^st^ factor: knowledge sharing & spontaneous communication 2^nd^ factor: focused communication	410.61*	26	15.79	50.18*	1	0.85	0.78	0.15	0.07
1^st^ factor: focused communication & knowledge sharing 2^nd^ factor: spontaneous communication	202.76*	26	7.79	113.19*	1	0.93	0.91	0.10	0.04
Three-factor model	87.00*	24	3.63	75.99*	2	0.99	0.96	0.06	0.03

*p < 0.001. The chi-square change was calculated by a scaling correction because the chi-square values for MLR cannot be compared directly (Muthén and Muthén, 2017). All two factor models were compared to the one-factor model. The three-factor model was compared to the best fitting two-factor model (i.e., 1^st^ factor: spontaneous communication; 2^nd^ factor: focused communication & knowledge sharing).

*Team communication* was measured using the TCS with the nine items used in Study 3, divided into three dimensions focused communication, knowledge sharing and spontaneous communication.

To assess *team planning* we used five items from Fisher (2014). An example item is “*My team sets goals for completing the task*.” The scale ranged from 1 (*strongly disagree*) to 5 (*strongly agree*). The scale originally had 6 items, but based on the analysis of the internal consistency of the scale, we excluded the item “*My team spends a lot of time discussing how to go about the task*” from aggregated scale scores.

*Time-spatial flexibility* was assessed with four items by Shockley and Allen (2007). Item examples are “*I vary my work schedule*” and “*I change my place of work so that it is adapted to my personal preferences and needs*.” Items were rated on a 5-point Likert scale from 1 (*completely disagree*) to 5 (*completely agree*).

*Collective efficacy* was measured with four items from Salanova et al. (2003). An example item is “*My group is totally competent to solve the task*.” Items were rated on a 5-point Likert scale from 1 (*strongly disagree*) to 5 (*strongly agree*).

*Team viability* was measured by one item from Ortega et al. (2010): “*If I would have the choice of working on this team again, I would do it*,” rated on a 5-point Likert scale from 1 (*strongly disagree*) to 5 (*strongly agree*).

#### Results

To examine the *factor structure* of the TCS on the team level, we conducted a CFA using Mplus 8 (Muthén and Muthén, 2017). Bartlett’s test of sphericity (*χ*^2^ (253) = 8103.195, *p* < 0.001) and the KMO measure of sampling adequacy (KMO = 0.912) of all used items was adequate, thus supporting the decision to continue with the CFA (Hair et al., 1995). Since this sample was clustered into teams, we used the “COMPLEX” analysis method, as it accounts for the nonindependence of individual observations within teams (Muthén and Muthén, 2017). The three-factor model showed a good fit to the data with *χ*^2^_24_ = 87.00, CFI = 0.98 and TLI = 0.96, RMSEA = 0.06 and SRMR = 0.03 (Bentler, 1992; Hu and Bentler, 1999; Vandenberg and Lance, 2000). To further assess the model fit, we compared the three-factor model with a one-factor model and three two-factor models – see Table 5 (Byrne, 2012). The change in *χ*^2^ was significant between consecutive models and the CFI difference between the best fitting two-factor model and our proposed three-factor model was higher than 0.01 and thus considered as relevant (Cheung and Rensvold, 2002). The data support Hypothesis 1 and show that the proposed TCS items load on three different factors. See Table 6 for an overview.

**Table 6:** Study 4: Final item wording, descriptive statistics, Cronbach’s alphas and standardized factor loadings from confirmatory factor analysis (team level).

Code	Item	Study 4				
		*M*	*SD*	Latent factor			1	2	3
*Focused communication*		3.98	0.46			
FC1	… we communicate in a timely manner.	4.07	0.87	0.80		
FC2	… we communicate exactly and precisely.	3.85	0.87	0.86		
FC3	… we communicate result-oriented.	3.97	0.88	0.83		
*Knowledge sharing*		4.24	0.42			
KS1	… we keep each other posted.	4.29	0.77		0.78	
KS2	… we know about the work of other team members.	4.21	0.78		0.79	
KS3	… we know the expertise of other team members.	4.15	0.83		0.83	
*Spontaneous communication*		4.18	0.42			
SC1	… we come up with solutions through spontaneous conversations.	4.01	0.87			0.78
SC2	… we also discuss things spontaneously.	4.22	0.84			0.89
SC3	… we communicate on a short notice.	4.26	0.78			0.77

To assess the *convergent*, *discriminant* and *predictive validity* of the TCS, individual scores had to be aggregated to their team means. To justify this aggregation, we conducted a one-way analysis of variance (ANOVA) using SPSS Version 27 (IBM Corp, 2020) to ascertain if there was a significant variation across teams. For all measures, there was a significant (*p* < 0.01) difference across teams: focused communication, *F*(129, 547) = 2.18, knowledge sharing, *F*(129, 547) = 2.15, spontaneous communication, *F*(129, 547) = 2.04, team planning, *F*(129, 547) = 2.01, team flexibility, *F*(129, 547) = 3.00, collective efficacy, *F*(129, 547) = 2.38 and team viability, *F*(129, 547) = 2.26. Based on the ANOVA results, we, then, calculated the intra-class correlation coefficients, ICC(1), which measures interrater reliability, and ICC(2), which estimates the reliability of the team mean (Bliese, 2000). All measures had acceptable levels of ICC(1) and ICC(2) – see Table 4 – hence we aggregated the data to the team level and continued with the analysis.

Campbell and Fiske (1959) proposed two aspects that should be considered to assess construct validity: the convergent validity (i.e., the degree of confidence we have that a trait is well measured by its indicators) and discriminant validity (i.e., the degree to which measures of different traits are unrelated). For convergent validity, average variance extracted (AVE) should be higher than 0.50 (Hair et al., 2010) and for discriminant validity, the square root of AVE of a scale should be higher than the scale’s correlations with other scales (Fornell and Larcker, 1981). We conducted a CFA with all six scale-based measures and used the standard factor loadings to compute AVE. The model showed a good fit with the data with *χ*^2^_194_ = 437.28, CFI = 0.96, TLI = 0.96, RMSEA = 0.04 and SRMR = 0.03. Further, all measures met the above-mentioned recommended values for AVE, apart from team planning where AVE = 0.41 (see Table 4). However, in cases where AVE is less than 0.50, but composite reliability (i.e., Cronbach’s alpha) is higher than 0.60, the convergent validity of the construct is still adequate (Fornell and Larcker, 1981).

To further assess the convergent and discriminant validity of the TCS, we analyzed the correlations of the three dimensions with team planning and team flexibility, respectively. We also conducted a Bonferroni-correction of the α level and divided the threshold for significance by the number of correlations that were tested for each hypothesis (i.e., 0.05/3 = 0.0167). When testing Hypothesis 2a, 2b and 2c, all TCS dimensions correlated with team planning at *p* < 0.01, thus supporting our assumption that team communication is related to team planning. For Hypothesis 3a, 3b and 3c, we assessed the discriminant validity of the TCS and looked at the correlations of each dimension with team flexibility. None of the correlations were significant, supporting discriminant validity.

To test for the *predictive validity* of the TCS, we conducted a multiple regression on the aggregated team data to assess the effect of TCS on collective team efficacy and team viability (see Table 7). For *collective efficacy*, the model was significant, explaining 52% of the overall variance (adj. *R*^2^ = 0.52, *F*(3, 129) = 47.42, *p* < 0.01), with all three factors being significant predictors and thus supporting Hypothesis 4a, 4b and 4c. For *team viability*, the model was also significant, explaining 52% of the overall variance (adj. *R*^2^ = 0.52, *F*(3, 129) = 47.08, *p* < 0.01). However, contrary to our expectations, only the dimensions *focused communication* (Hypothesis 5a) and *knowledge sharing* (Hypothesis 5b) were significant predictors of team viability. Thus, we did not find support for Hypotheses 5c that spontaneous communication is positively related to team viability.

**Table 7:** Coefficients of multiple linear regression of TCS on team collective efficacy.

	*b*	*S.E.*	*β*	*t*	*p*
*Collective efficacy*					
(Intercept)	1.98	0.23		8.87	0.00
Focused communication	0.19	0.06	0.28	2.94	0.00
Knowledge sharing	0.24	0.08	0.30	2.98	0.00
Spontaneous communication	0.19	0.07	0.24	2.96	0.00
*Team viability*					
(Intercept)	1.25	0.29		4.24	0.00
Focused communication	0.27	0.09	0.29	3.12	0.00
Knowledge sharing	0.43	0.11	0.41	4.03	0.00
Spontaneous communication	0.10	0.09	0.09	1.12	0.26

## Discussion

The goal of this paper was to develop, refine and validate a scale that captures the behavioral components of communication in teams, an aspect neglected in the literature to date. We draw on relational coordination theory (Gittell, 2000, 2011a,b) and literature from the remote work context (Hinds and Mortensen, 2005; Methot et al., 2021; Jämsen et al., 2022) as well as (group) interview data with workers using time-spatial flexibility and propose the following three dimensions of communication to be particularly relevant in flexible teams: (1) focused communication, (2) knowledge sharing and (3) spontaneous communication. Results from confirmatory factor analysis on the individual as well as the team-level supported the three-factor structure.

The results of the validation with flexible teams showed support for convergent as well as discriminant validity. The three dimensions of the TCS are associated with a similar measure (i.e., team planning), but are at the same time distinctive from a different measure (i.e., time-spatial flexibility). Moreover, the results showed that the TCS predicts important team outcomes providing evidence for predictive validity. All three TCS dimensions were found to significantly predict collective efficacy showing that team communication influences team’s confidence in future success. Our results reveal that both sharing information and expertise between the team members (i.e., knowledge sharing), but also the way this information is shared (i.e., focused and spontaneous communication) are important factors for team members’ beliefs about their capability to perform the tasks. This is in line with previous research that highlights the beneficial effect of a high quality of communication in interdependent teams (Gittell, 2000, 2011a,b, 2016) and particularly in flexible teams (Shockley et al., 2021).

With regard to the second outcome, our results reveal that team viability was significantly predicted by focused communication and knowledge sharing, but not by spontaneous communication. Thus, in our sample spontaneous communication does not determine the willingness of team members to stay in the team. However, spontaneous communications about non-task related aspects with colleagues were recently shown to be relevant for building positive emotions at work (Methot et al., 2021) and also fostering belongingness (Puranik et al., 2021). Thus, we have the following explanations why we could not find a positive relationship between spontaneous communication and team viability: First, data collection took place during and in between the COVID-19 lockdowns and spontaneous communication has been considerably impaired due to the high prevalence of remote work (Jämsen et al., 2022). Since spontaneous communication was missing in many teams during that time, changing the team might not be considered as solution to overcome this lack and therefore did not predict team viability. An alternative explanation for the non-significance might be that the association between spontaneous communication and team viability is moderated by other factors such as team leaders’ behavior. In teams, where leaders managed to compensate for lack of spontaneous communication during the COVID-19 lockdowns, spontaneous communication might not influence team members’ willingness to stay with the team. However, in teams where such a compensation did not occur, the lack of spontaneous communication might be related to lower levels of team viability.

### Contribution of the TCS

The TCS scale encompassing focused communication, knowledge sharing and spontaneous communication contributes to existing team communication measures in numerous ways. First and most important, we address a neglected area in work and organization research by focusing on team communication behaviors. Previous measures of team communication have either focused on frequency (of communication, Bunderson and Sutcliffe, 2003; or of knowledge sharing, Kessel et al., 2012), or on assessing satisfaction with team communication (i.e., Hoegl and Gemuenden, 2001) and personal attitudes toward teamwork (i.e., Pollard et al., 2004; Cooper et al., 2020). Communication frequency, however, was shown to be less relevant for team outcomes than the quality of communication (Shockley et al., 2021) and could also result in exhaustion due to information overkill. With regard to the perceived quality of communication, we argue that this is rather the emergent state following communication behaviors and the usage of such measures provides little knowledge about how to improve the quality of communication in the actual team. By specifically addressing communication behaviors, concrete improvements can be derived from our measures. Furthermore, using the team as the reference point for the communication behaviors represents an important added value in contrast to several existing scales (Pollard et al., 2004; Kessel et al., 2012; Cooper et al., 2020) as it provides justification for aggregated scores (Klostermann et al., 2021).

A second contribution is that the TCS takes into account the increased prevalence of time-spatial flexibility in contemporary work teams. Although the items of the TCS are formulated so generally that the scale can be used in any work team to assess team communication, it aimed to take into account the communication dimensions being most challenged and relevant in flexible teams. In order to do so, we built upon a sound theoretical background (relational coordination theory; Gittell, 2000, 2002), but also on different sources of empirical data (quantitative and qualitative), which were gathered from employees working in flexible teams. We consider the suitability for flexible teams as particularly important because working remotely has become the new normal for office workers across the globe.

Finally, the third contribution of the TCS scale is that it encompasses a dimension, which has been neglected so far in existing team communication measures, or it has been measured only as frequency of spontaneous exchanges among team members (Hinds and Mortensen, 2005). Spontaneous communication is a rather under-researched field. Maybe because this type of unplanned and informal communication did not require particular attention in the co-located work setting. However, the recent experiences due to the high intensity of remote work drew the attention onto this topic as a lack thereof emphasized its importance (Jämsen et al., 2022). Empirical evidence shows that daily small talks enhance positive emotions and well-being at work (Methot et al., 2021) and also foster belongingness despite its potential intruding character (Puranik et al., 2021). In line with this research, our current findings also suggest that spontaneous communication can predict important team outcomes such as collective efficacy. Therefore, including the assessment of spontaneous communication behaviors is an important asset and added value of the TCS.

### Limitations and suggestions for future research

Our study presents several limitations that can be addressed in future research. First of all, the results are obtained based on cross-sectional data which limits the scope of predictive conclusions. Additionally, all collected data were based on self-reports, therefore common method bias may have influenced the results. Future research should therefore longitudinally explore the effects of TCS for important team outcomes over time.

Second, with regard to predictive validity, our results revealed that all three dimensions of the TCS determine perceived collective efficacy. However, to fully understand the role of communication for team performance, other (more objective) indicators should be used in future research. Also, the dimension spontaneous communication did not relate to team viability. Thus, research is needed that investigates potential moderators of the association between communication and team outcomes. The “forced” remote work due to the COVID-19 pandemic might have had an influence and therefore it is necessary to explore this relationship further in post-pandemic studies.

Third, although the TCS was developed to particularly capture the context of flexible teams, it is applicable for other team contexts as well. More knowledge is needed about the impact of this specific context. Therefore, future research should compare how the dimensions of the TCS differ in fully virtual, flexible or fully co-located teams. Since recent communication studies point toward the need of culture-specific scales (e.g., Tkalac Verčič and Špoljarić, 2020), it could be of interest to investigate the validity of the TCS scale across different cultures. Furthermore, it could be of interest to examine the TCS scale in additional sectors with interdependent teams, such as healthcare, public service or in non-profit organizations.

## Practical implications

Research shows that communication has changed recently due to the boost in remote working: it has become more static and siloed and the extent of synchronous communication has decreased, which makes it harder for remote workers to share information (Yang et al., 2021). However, relational coordination theory (Gittell et al., 2010) argues that behavioral components of communication also define relationship aspects and are key for team performance (Gittell, 2016). Therefore, team leaders of flexible teams could foster social relations and bonds between team members by deliberately managing team communication behaviors. For example, it is crucial to define times and places where remote workers can meet, exchange knowledge and engage in spontaneous (informal) communication. Moreover, to ensure that information is shared across all team members, leaders should establish communication guidelines that ensure a timely and focused communication with regard to team tasks. In addition to team leaders’ behavior, team members should be empowered and trained to adequately communicate with their colleagues. This also includes sensitizing about the relevance of knowledge sharing as well as spontaneous communication that might not immediately address task-related aspects. Finally, the TCS can be used as a tool for team development. It can serve as a screening tool to reflect on teams’ communication behaviors.

## Conclusion

The TCS is a questionnaire-based instrument to measure the behavioral aspects of team communication encompassing focused communication, knowledge sharing, and spontaneous communication. Building upon theory as well as empirical data, it is suitable for use in all contexts and for all teams (co-located, virtual, flexible), but especially for teams that work flexibly (teams where employees can vary work time and place) as it captures aspects of communication relevant for today’s team work characterized by high unpredictability. In contrast to previous measures, it takes into account spontaneous communication in teams, which has been found to be impaired in teams during the COVID-19 pandemic, but is particularly important for relational communication (Methot et al., 2021; Puranik et al., 2021; Jämsen et al., 2022). In addition to its value in team research, having a psychometrically sound team instrument enables practitioners to use the TCS as a tool for team development in order to identify areas of improvement for team communication.

## Data availability statement

The raw data supporting the conclusions of this article will be made available by the authors, without undue reservation.

## Ethics statement

Ethical review and approval was not required for the study on human participants in accordance with the local legislation and institutional requirements. The patients/participants provided their written informed consent to participate in this study.

## Author contributions

MH-T: initial idea to develop the scale, planning and execution of all studies, conduction of interviews or supervising theses of students who conducted interviews and group discussions, generation of item pool, analysis and interpretation of data, writing—original draft preparation and review, and editing. IL: in-depth analysis of data and writing-up results, writing—original draft preparation and review, and editing. SH: planning and execution of study 4, interpretation of data, writing—draft and critical revisions preparation. BK: planning and execution of study 4, interpretation of data, and critical revisions. All authors contributed to the article and approved the submitted version.

## Funding

Transcription of 31 interviews was financed by the organization under study to obtain knowledge about their coordination processes. Study 1 was financed by ATP architekten ingenieure. Study 2 and Study 3 were conducted in cooperation with the Chamber of Labour of Lower Austria, which organized and financed the distribution of the postal questionnaires to the participants. Study 4 was funded in part by the Austrian Science Fund (FWF) P29408-G29. The funders were not involved in the study design, collection, analysis, interpretation of data, the writing of this article or the decision to submit it for publication.

## Conflict of interest

The authors declare that the research was conducted in the absence of any commercial or financial relationships that could be construed as a potential conflict of interest.

## Publisher’s note

All claims expressed in this article are solely those of the authors and do not necessarily represent those of their affiliated organizations, or those of the publisher, the editors and the reviewers. Any product that may be evaluated in this article, or claim that may be made by its manufacturer, is not guaranteed or endorsed by the publisher.
