# Supplementary material for: Development and validation of a scale to measure team communication behaviors
Source: Front Psychol. 2022 Dec 8;13:961732. doi: 10.3389/fpsyg.2022.961732 (PMC9775872; doi:10.3389/fpsyg.2022.961732)
Supplement: Supplementary file 1 [file Data_Sheet_1.docx]

Supplementary Material

**Table S1. English Version of the TCS**

In our team…

| *Factor* | *Code* | *Item Wording* |
| --- | --- | --- |
| Focused | FC1 | ... we communicate in a timely manner. |
| Communication | FC2 | ... we communicate exactly and precisely. |
|  | FC3 | ... we communicate result-oriented. |
| Knowledge | KS2 | ... we keep each other posted. |
| Sharing | KS3 | ... we proactively inform the others about relevant news. |
|  | KS5 | *...* we exchange our knowledge and experiences. |
| Spontaneous | SC1 | ... we come up with solutions through spontaneous conversations. |
| Communication | SC2 | ... we also discuss things spontaneously. |
|  | SC3 | ... we communicate on a short notice. |

*Note.*

## Answer scale: 1 = strongly disagree, 2 = somewhat disagree, 3 =  neither disagree nor agree, 4 = somewhat agree, 5 = strongly agree

**Table S2. German Version of the TCS**

Bei uns im Team …

| *Factor* | *Code* | *Item Wording* |
| --- | --- | --- |
| Focused | FC1 | ... kommunizieren wir zeitgerecht |
| Communication | FC2 | ... kommunizieren wir genau und präzise. |
|  | FC3 | ... kommunizieren wir ergebnisorientiert. |
| Knowledge | KS2 | ... halten wir uns über unsere Arbeit auf dem Laufenden. |
| Sharing | KS3 | … geben wir relevante Informationen proaktiv weiter. |
|  | KS5 | … tauschen wir unsere Erfahrungen und unser Wissen aus. |
| Spontaneous | SC1 | … kommen wir durch ungeplante, spontane Kommunikation zu Lösungen. |
| Communication | SC2 | … besprechen wir Dinge auch spontan. |
|  | SC3 | … tauschen wir uns kurzfristig miteinander aus. |

*Note.*

## Answer scale: 1 = trifft gar nicht zu, 2 = trifft eher nicht zu, 3 =  teils, teils, 4 = trifft eher zu, 5 = trifft völlig zu
